# Supplementary figures and images for: Development of SNP Markers for White Immature Fruit Skin Color in Cucumber (Cucumis sativus L.) Using QTL-seq and Marker Analyses
Source: Plants (Basel). 2021 Oct 29;10(11):2341. doi: 10.3390/plants10112341 (PMC8625156; doi:10.3390/plants10112341)

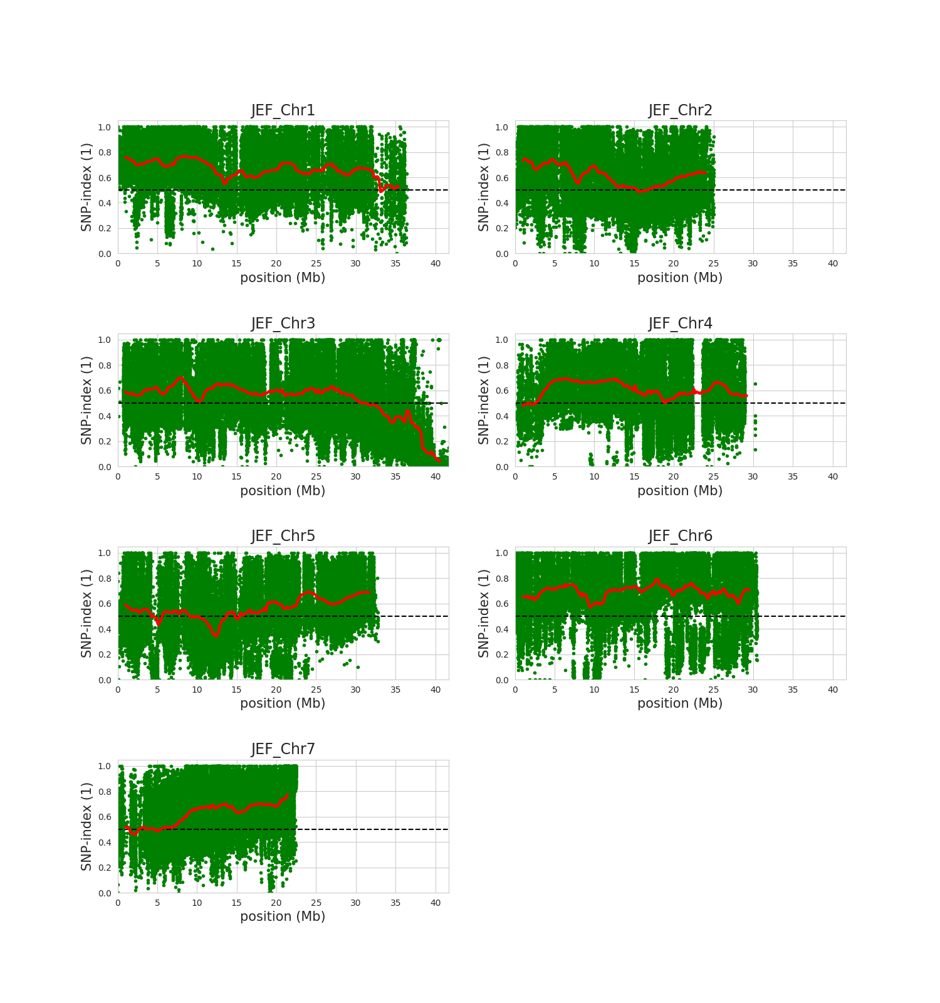

Supplement: Supplementary file 1 [file plants-10-02341-s001.zip › Figure S1.tif]

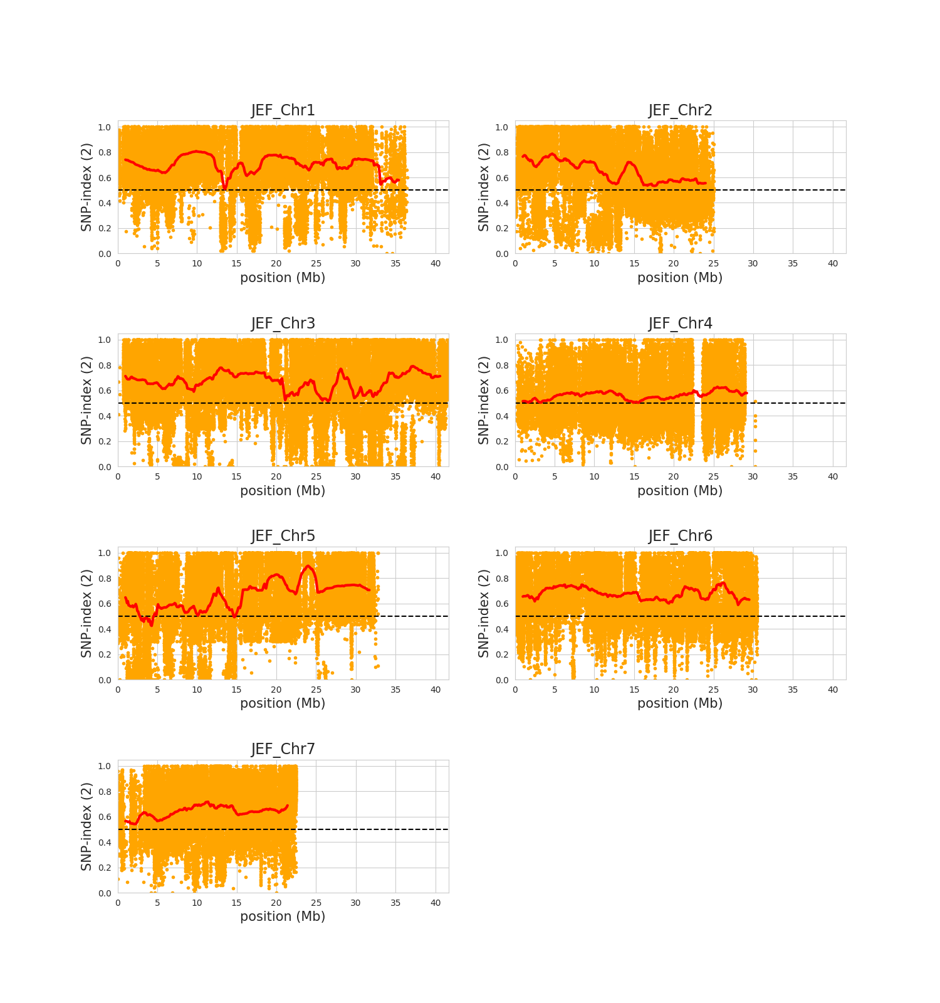

Supplement: Supplementary file 1 [file plants-10-02341-s001.zip › Figure S2.tif]

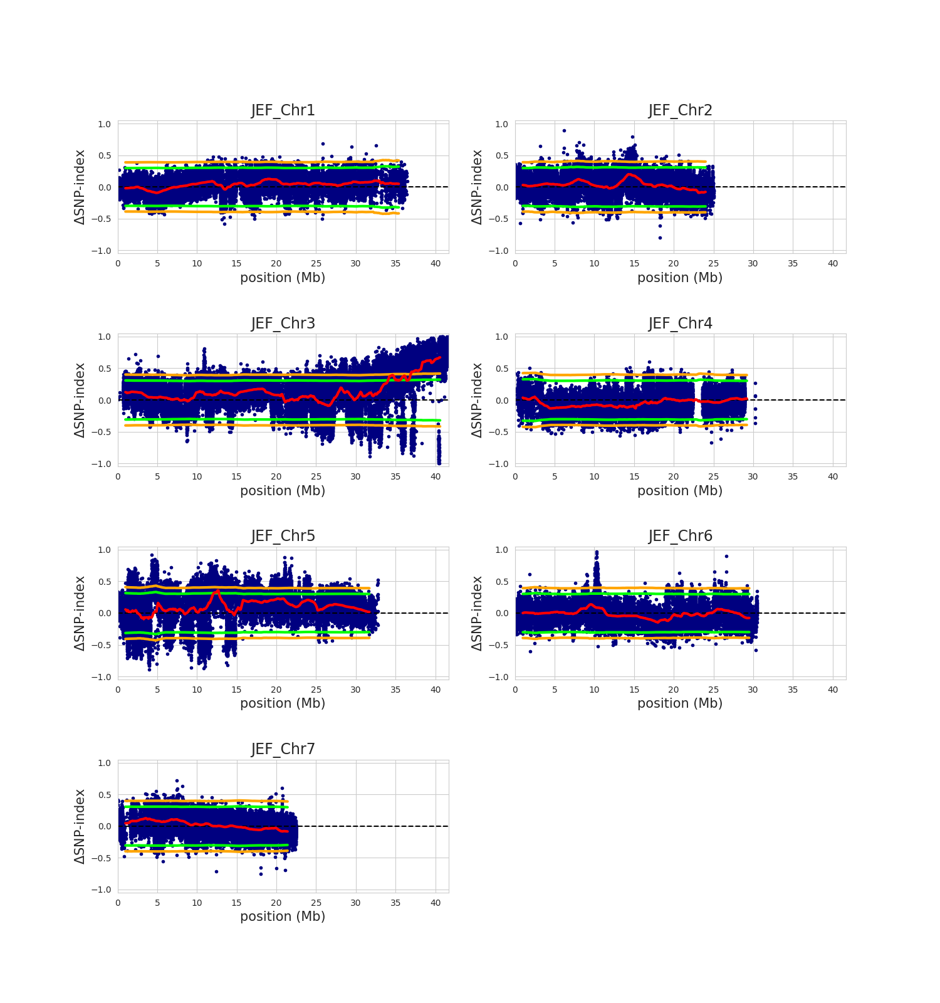

Supplement: Supplementary file 1 [file plants-10-02341-s001.zip › Figure S3.tif]

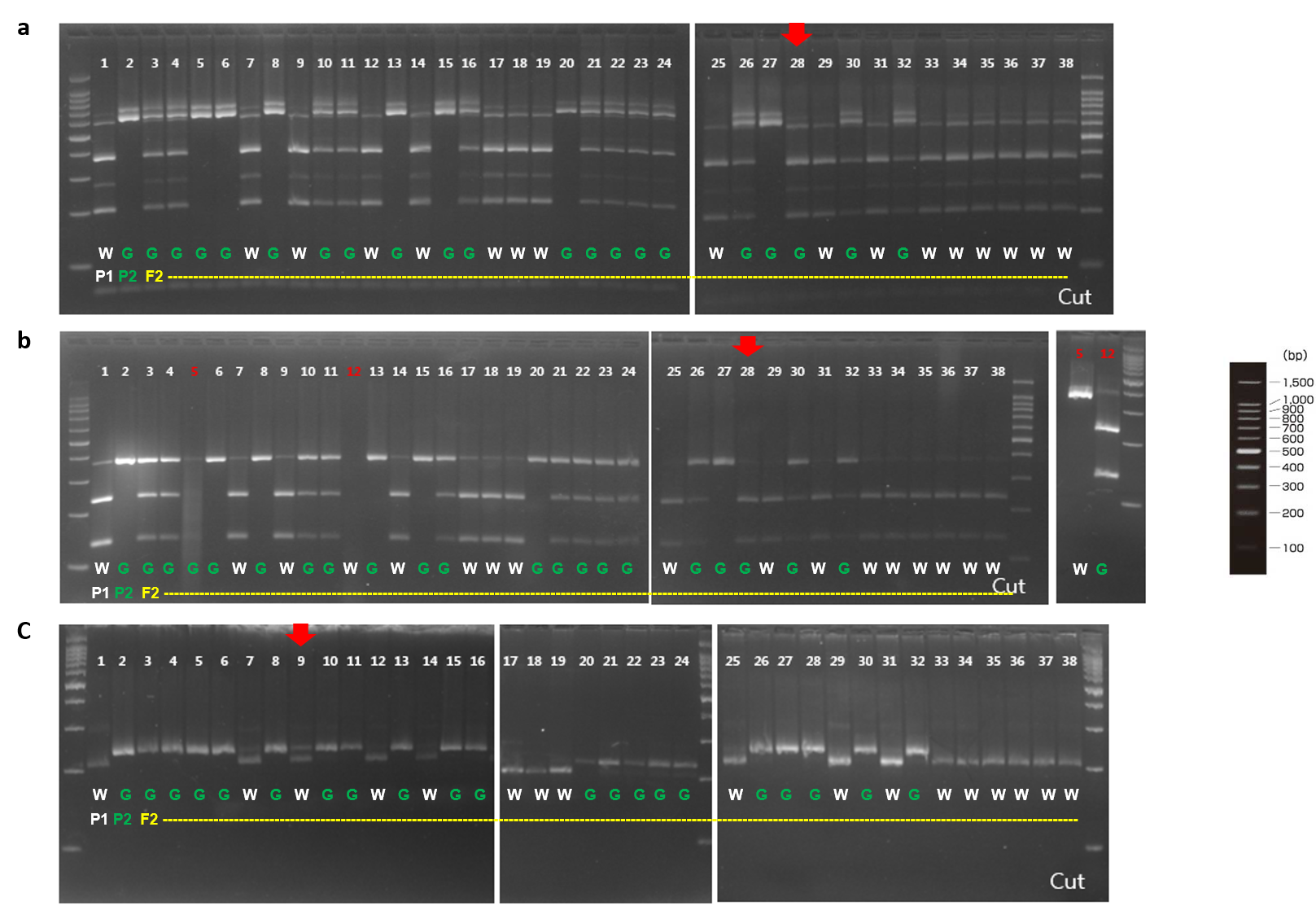

Supplement: Supplementary file 1 [file plants-10-02341-s001.zip › Figure S4.tif]
